# Comparative Analysis of Type 2 Diabetes Prevalence and Clinical Profiles in Ethiopia and Nigeria

**DOI:** 10.1111/1753-0407.70078

**Published:** 2025-04-02

**Authors:** Assefa Mulu Baye, Teferi Gedif Fenta, Suvi Karuranga, Ifeyinwa Dorothy Nnakenyi, Ekenechukwu Esther Young, Colin Palmer, Ewan R. Pearson, Ifeoma Isabella Ulasi, Adem Y. Dawed

**Affiliations:** ^1^ Department of Pharmacology and Clinical Pharmacy College of Health Sciences, Addis Ababa University Addis Ababa Ethiopia; ^2^ Department of Pharmaceutics and Social Pharmacy College of Health Sciences, Addis Ababa University Addis Ababa Ethiopia; ^3^ European Society for Emergency Medicine Antwerp Belgium; ^4^ Department of Chemical Pathology College of Medicine, University of Nigeria & University of Nigeria Teaching Hospital Enugu Nigeria; ^5^ Department of Medicine College of Medicine, University of Nigeria & University of Nigeria Teaching Hospital Enugu Nigeria; ^6^ Division of Population Health and Genomics Ninewells Hospital and School of Medicine, University of Dundee Dundee UK; ^7^ Department of Internal Medicine Alex Ekwueme Federal University Teaching Hospital Abakaliki Nigeria

**Keywords:** clinical characteristics, diabetes, oral glucose tolerance test, sub‐Sahara Africa

## Abstract

**Aims:**

We aimed to determine the prevalence of diabetes and the clinical profiles of Type 2 diabetes in Ethiopia and Nigeria.

**Methods:**

A community‐based cross‐sectional study was carried out from November 01, 2020 to October 21, 2021 among 1727 participants using a multistage sampling method. The WHO's STEPs tool was employed. Both fasting and oral glucose tolerance tests were used for screening and American Diabetes Association's (ADA's) diagnostic criteria was used. Stata version 17 was used for analysis. Analysis of Variance (ANOVA) and the Chi‐square test were used to compare variables. Significance was declared at a *p*‐value less than 0.05.

**Result:**

Of the surveyed participants, 872 (50.5%) were men and the mean age was 44.6 years. The overall prevalence of prediabetes and diabetes was 15.8% and 7.0%, respectively. Impaired fasting glycaemia and glucose tolerance were 11.8% and 11.5%, respectively. The prevalence of newly diagnosed diabetes was 3.4% by fasting and 4.0% by oral glucose tolerance test. Participants with normal blood glucose were younger and had a lower weight, body mass index (BMI), waist circumference, diastolic, and systolic blood pressure, and history of hypertension than those with prediabetes and diabetes.

**Conclusion:**

In the present study, there is a notably high diabetes and prediabetes prevalence in the study settings. Individuals with diabetes in Ethiopia compared to Nigeria and the West have different anthropometric and clinical profiles characterized by a young age of onset, leanness, lower BMI, and waist circumference. Hence, the management of diabetes shall be tailored to the unique physiologic and clinical profiles of the population.

## Graphical abstract

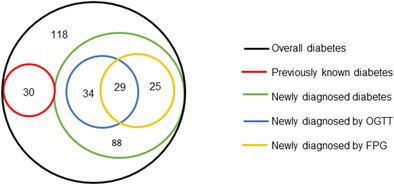

**Summary**

- We found that Ethiopian individuals with diabetes had a different anthropometric and clinical profile characterized by a young age of onset, leanness, lower BMI, and waist circumference compared to Nigerians and the west.
- This study highlights the imperative need for further research and careful considerations when formulating therapeutic strategies for diabetes specifically tailored to the distinct physiological and clinical characteristics of populations in Ethiopia, Nigeria, and Sub‐Saharan Africa at large.

## 1 Introduction

Diabetes is a chronic disorder characterized by elevated blood glucose levels, caused by either inadequate insulin production in the pancreas or resistance to insulin [1]. It poses a significant worldwide health challenge, accounting for more than half a billion cases (10.5% of the world's adult population) and 6.7 million deaths among adults in 2021 [2] projected to rise to 643 million by 2030.

The number of individuals with diabetes in Sub‐Sahara Africa (SSA) was about 24 million by 2021, with the highest proportion of undiagnosed diabetes cases (53.6%) as compared to high‐income countries (28.8%) [2]. According to the report from the International Diabetes Federation (IDF) [2], Nigeria and Ethiopia hold the second and fourth positions, respectively, in terms of the highest number of people with diabetes in SSA.

Guidelines recommend the early detection of diabetes to achieve adequate plasma glucose control and delay the onset of complications [3, 4]. Both the American Diabetes Association (ADA) and IDF recommend fasting plasma glucose (FPG) and the 2‐h postprandial oral glucose tolerance test (OGTT) to identify prediabetes and diabetes [4, 5]. These methods come with distinct advantages and disadvantages in practical use. In healthcare settings, FPG is preferred for identifying diabetes in high‐risk groups due to its convenience and cost‐effectiveness compared to OGTT. However, it should be noted that OGTT surpasses FPG in sensitivity, diagnosing a greater number of individuals with prediabetes and diabetes [6], despite being less feasible for mass screening [5].

According to a recent systematic review and meta‐analysis, the prevalence of Type 2 diabetes in SSA varied from 1.4% to 22.5% [7]. There are concerns about the accuracy of these findings due to the study methods used, such as sampling strategies and study settings. Moreover, studies relied on less reliable self‐report, random, or fasting capillary blood samples, often conducted in health facilities, which may affect the overall reliability of the results [8].

In order to develop effective and context‐specific diabetes interventions, it is crucial to understand the unique sociodemographic and clinical profiles of affected populations. Contrary to the western population, diabetes has also been reported to be common among young and non‐overweight individuals, especially among Asian and SSA countries [9]. Exploring the variability in sociodemographic and clinical characteristics of people with diabetes within SSA provides a foundation for effective interventions. But evidence on the anthropometric, socio‐demographic, and clinical profile of diabetes is limited to formulate tailored recommendations for prevention, screening, diagnosis, and treatment of diabetes in SSA.

Thus, there is a need for high‐quality population‐based studies in SSA to obtain more accurate and comprehensive data on the prevalence of Type 2 diabetes. This research aimed also to produce population‐based evidence on the prevalence of diabetes and prediabetes among participants from the two most populous countries in SSA, Ethiopia and Nigeria. Moreover, this paper investigates the heterogeneity of the clinical phenotypes of type 2 diabetes in SSA, using examples from two populations on the continent.

## 2 Methods

### 2.1 Study Design

The Diabetes Epidemiologists' Network in Sub‐Saharan Africa (DENSSA) represents a collaborative research initiative involving the University of Dundee in the United Kingdom, Addis Ababa University in Ethiopia, and the University of Nigeria in Nigeria. A community‐based cross‐sectional study was carried out from November 01, 2020 to October 21, 2021.

### 2.2 Survey Population

The study was conducted using a population from the Butajira Rural Health Program (BRHP) in central Ethiopia and Enugu State district in Nigeria. BRHP consisted of one semi‐urban and nine rural kebeles, which represent the smallest administrative units in Ethiopia.

### 2.3 Sample Size Determination and Sampling Procedure

Sample size was determined using the single population proportion formula, using a 3.9% prevalence of diabetes for SSA [10], *d* = 1.25% for better precision, and considering a design effect of two for complex multistage design. Anticipating a 10% non‐response, about 1000 individuals aged ≥ 18 years from each of the two study sites were included.

We employed a multistage sampling method to choose the participants for the study. For the Ethiopian study, six kebeles (smallest administrative unit) were selected randomly out of 10 kebeles that are part of the BRHP, whereas three Local Government Areas (LGAs)—Enugu North, Enugu West, and Nkanu West in Enugu State—were selected in Nigeria. A proportional sampling method was used to select households from each administrative unit. A randomly selected adult was invited to participate in the study. In households with multiple eligible individuals, a single person was randomly chosen.

### 2.4 Inclusion and Exclusion Criteria

This study included adults who were 18 years or older and provided their consent to participate. Individuals who did not fast for at least 8 h, pregnant women, and those who were taking medications such as corticosteroids, atypical antipsychotics, and protease inhibitors were excluded from the study. Individuals with a history of diabetes were also not included for the OGTT.

### 2.5 Survey Instrument

The study used the WHO's STEPwise approach to NCD risk factor surveillance (STEPS) instrument version 3.1 to assess diabetes risk factors [11].

### 2.6 Data Collection Procedure

Data collectors were trained on study objectives, survey tools, physical measurements, and blood sampling collection and testing. Tools were pre‐tested on 25 participants from each site to refine the questionnaire's structure, flow, and interview time before data collection. Local guides (health workers, volunteers) aided in reaching households. On the first day for STEP I data collection, households were visited, and the aim of the survey was explained. Then respondent's demographic and socioeconomic status; tobacco, alcohol, and Khat use; diet, including fruit, vegetable, dietary salt intake; physical activity; history of raised blood pressure, diabetes, raised cholesterol and/or CVDs, and lifestyle advice received were collected. Physical measurements (STEP 2) including blood pressure (BP), heart rate, weight, height, waist, and hip circumference, and laboratory tests (STEP 3) were taken on the next day after the questionnaire survey. Blood pressure and heart rate were taken twice on the right arm while seated, using a Folee DX‐B1 monitor (Jiangsu Folee Medical Equipment, China). If BP readings differed by > 5 mmHg, a third was taken and the mean of two (or three) readings was used. Blood samples were collected from participants who had fasted for at least 8 h at nearby health facilities.

Capillary blood glucose level was determined using Fia Biomed Precisa blood glucose monitoring system (Fia Biomed GmbH, Emsdetten, Germany). Participants with a capillary fasting glucose level ≥ 126 mg/dL were referred to the nearby health center. For those with capillary blood glucose level < 180 mg/dL, two blood samples, 4 mL before and 2 mL after the 75 g oral glucose drink, were collected. The fasting blood and the 2‐h post glucose load samples were collected into two 2 mL fluoride oxalate tubes (gray top). OGTT was performed using a standard solution, Polycal Liquid 200 mL (Nutricia Ltd., Wiltshire, UK).

### 2.7 Diagnostic Criterion

The definition of the ADA is used to identify diabetes and pre‐diabetes cases [4]. The results of plasma glucose testing are classified as follows: isolated impaired fasting glucose (IFG) is defined as plasma glucose levels between 100 mg/dL and 125 mg/dL, with a 2‐h PG of < 140 mg/dL. Isolated impaired glucose tolerance (IGT) is defined as a glucose level < 100 mg/dL of fasting and a 2‐h PG between 140 mg/dL and 199 mg/dL. Newly diagnosed diabetes is defined as a FPG level of ≥ 126 mg/dL or 2‐h OGTT of ≥ 200 mg/dL or both. When people receive a medical diagnosis of diabetes from healthcare providers previously, it is regarded as a known diabetes case.

### 2.8 Data Quality Assurance

The items of the tool were translated into local languages, then translated back to English to assure consistency and accuracy. Based on the results of the pre‐test and back translation, the questionnaires were further reviewed and completed. Field data supervisors carried out intensive monitoring and follow‐up during each phase of data collection.

### 2.9 Statistical Analysis

Data analysis was conducted on Stata V.17 software program (StataCorp. Stata Statistical Software: Release 17. College Station, TX: Stata Corp LP). Estimates of continuous variables were summarized as mean (SD) and frequencies and proportions (95% CI) for categorical variables. Comparison of means of quantitative variables across different diabetes categories was done by analysis of variance. Association between categorical variables was checked using chi‐square testing. Statistical significances were tested at *p* < 0.05.

## 3 Results

### 3.1 Demographic Information Results

Among the 2000 planned samples, 1727 individuals were contacted and given consent for the survey, making the overall response rate 86.4%. The rest refused to provide blood samples. The characteristics of individuals who refused to provide blood samples were not significantly different from those who provided blood samples, as shown in Supporting Information (Data S1).

Out of the entire survey population, 872 individuals (50.5%) were men. The mean age of the participants was 44.6 years, with Ethiopians having a mean age of 41.0 years and Nigerians having a mean age of 47.2 years. More than half of the respondents (54.3%) were under the age of 45, as presented in Table 1.

**TABLE 1:** Summary of sociodemographic information by country of residence, Ethiopia and Nigeria, 2021.

Characteristics	Country		Total, *n* (%)
	Ethiopia, *n* (%)	Nigeria, *n* (%)	
Sex			
Men	272 (37.3)	600 (60.2)	872 (50.5)
Women	458 (62.7)	397 (39.8)	855 (49.5)
Age, years			
Mean (SD)	41.0 (±13.62)	47.2 (±17.58)	44.6 (±16.31)
18–29	149 (20.4)	189 (19.0)	338 (19.6)
30–44	311 (42.6)	289 (29.0)	600 (34.7)
45–59	167 (22.9)	223 (22.4)	390 (22.6)
60–69	71 (9.7)	153 (15.3)	224 (13.0)
≥ 70	32 (4.4)	143 (14.3)	175 (10.1)
Current residence			
Urban^a^	455 (62.3)	534 (53.6)	989 (57.3)
Rural	275 (37.7)	463 (46.4)	738 (42.7)
Religion			
Christian	205 (28.1)	985 (98.8)	1190 (68.9)
Muslim	525 (71.9)	3 (0.3)	528 (30.6)
Traditional beliefs	—	9 (0.9)	9 (0.5)
Current marital status			
Never married	63 (8.6)	225 (22.6)	288 (16.7)
Currently married	570 (78.1)	635 (63.7)	1205 (69.8)
Widowed	60 (8.2)	131 (13.1)	191 (11.1)
Divorced/separated	37 (5.1)	6 (0.6)	43 (2.5)
Highest grade of education completed			
No formal education	293 (40.1)	168 (16.9)	461 (26.7)
Some primary education	212 (29.0)	53 (5.3)	265 (15.3)
Completed primary education	77 (10.5)	150 (15.0)	227 (13.1)
Junior secondary education	56 (7.7)	38 (3.8)	94 (5.4)
Senior secondary education	57 (7.8)	226 (22.7)	283 (16.4)
More than secondary education	35 (4.8)	362 (36.3)	397 (23.0)
Occupation			
Government employee	43 (5.9)	204 (20.5)	247 (14.3)
Non‐government employee	22 (3.0)	19 (1.9)	41 (2.4)
Self employed	9 (1.2)	261 (26.2)	270 (15.6)
Unpaid	656 (89.9)	513 (51.5)	1169 (67.7)

Abbreviation: SD: Standard deviation.

^a^ Semi‐urban for the Ethiopian study participants.

### 3.2 Social Drug Use

Among the surveyed participants, 6.7% were current smokers, while 27.4% of the study participants drank alcohol at least once within 30 days of the survey, as presented in Table 2.

**TABLE 2:** Pattern of substance use among all respondents by country of residence, Ethiopia and Nigeria, 2021.

Participant characteristics	Ethiopia	Nigeria	Total
Cigarette smoking			
Current smokers, *n* (%)	35 (4.8)	81 (8.1)	116 (6.7)
Daily smokers, *n* (%)	27 (3.7)	42 (4.2)	69 (4.0)
Former smokers, *n* (%)	23 (3.2)	46 (4.6)	69 (4.0)
Ever smoked, *n* (%)	58 (7.9)	127 (12.7)	185 (10.7)
Mean (SD) age of smoking started, years	28.9 ± 6.7	26.5 ± 7.7	27.2 ± 7.5
Mean (SD) duration of smoking, years	19.3 ± 13.1	14.1 ± 11.1	15.5 ± 11.8
Alcohol consumption, *n* (%)			
Current drinker	107 (14.7)	367 (36.8)	474 (27.4)
Drink in past 12 months, not current	129 (17.7)	473 (47.4)	602 (34.9)
Past 12‐month abstainer	494 (67.6)	157 (15.7)	651 (37.7)
Life time abstainer	567 (77.7)	544 (54.6)	707 (59.1)
Khat user, *n* (%)			
Current chewer	430 (58.9)	NA	430 (58.9)
Past chewer	37 (5.1)	NA	37 (5.1)
Never chewer	264 (36.2)	NA	264 (36.2)

Abbreviations: NA, not applicable; SD, standard deviation.

### 3.3 Dietary Patterns and Physical Activity

On average, participants consumed fruits 2.7 days and vegetables 4.6 days per week. In terms of servings per day, they had 1.3 servings of fruits and 1.9 servings of vegetables daily. Nearly one‐fourth (22%) were not meeting WHO's physical activity recommendations, as shown in Table 3.

**TABLE 3:** Dietary pattern and physical activity of participants by country of residence, Ethiopia and Nigeria, 2021.

Fruit and vegetables	Ethiopia	Nigeria	Total
Fruits			
Mean (SD) number of days fruits consumed in a weak	1.9 ± 1.0	3.4 ± 1.7	2.7 ± 1.6
Mean (SD) number of servings of fruits per day	1.5 ± 0.8	1.1 ± 0.5	1.3 ± 0.7
Vegetables			
Mean (SD) number of days vegetables consumed in a weak	4.3 ± 2.1	4.9 ± 1.7	4.6 ± 1.9
Mean (SD) number of servings of vegetables per day	2.4 ± 0.8	1.5 ± 0.7	1.9 ± 0.9
Salt^a^			
Add salt to food when cooking or preparing food, *n* (%)	508 (69.6)	113 (11.3)	621 (36.0)
Eat processed foods high in salt, *n* (%)	110 (15.1)	300 (30.1)	410 (23.7)
Quantity of salt consumption, *n* (%)			
Far too much or too much	108 (14.8)	19 (1.9)	127 (7.4)
Just the right amount	433 (59.3)	932 (93.5)	1365 (79.0)
Too little or far too little	189 (25.9)	46 (4.6)	235 (13.6)
Physical activity			
Not meeting WHO's recommendations, *n* (%)	173 (23.7)	205 (20.7)	378 (21.9)
Levels of physical activity ^b^, *n* (%)			
High level	374 (51.2)	621 (62.3)	995 (57.6)
Moderate level	162 (22.2)	243 (24.4)	405 (23.5)
Low level	194 (26.6)	133 (13.3)	327 (18.9)
Contribution of types of activities (%)			
Work‐related activity	63.6	55.7	59.0
Transport‐related activity	34.6	34.0	34.2
Recreational‐related activity	1.8	10.3	8.8

Abbreviations: SD, standard deviation; WHO, World Health Organization.

^a^ Self‐reported.

^b^ According to WHO, high‐level physical activity is vigorous‐intensity activity for at least 3 days per week, achieving at least 1500 MET‐min per week; or seven or more days of any combination of walking, moderate‐ or vigorous‐intensity activities achieving a at least 3000 MET‐min per week. Moderate‐level physical activity is meeting any of the following criteria: three or more days of vigorous‐intensity activity of at least 20 min per day; or five or more days of moderate‐intensity activity or walking for at least 30 min per day; or five or more days of any combination of walking, moderate‐ or vigorous‐intensity activities achieving at least 600 MET‐min per week. Low‐level physical activity is a person not meeting the criteria for moderate‐ or high‐level categories.

### 3.4 History Cardiovascular Disorders

Nearly half (49.2%) of the participants had never had their BP measured. Of those who had measurements, 22.2% were diagnosed with high BP, accounting for 11.3% of the study participants (Table 4).

**TABLE 4:** History cardiovascular diseases by country of residence, Ethiopia and Nigeria, 2021.

History	Ethiopia, % (95% CI)	Nigeria, % (95% CI)	Total, % (95% CI)
History of hypertension			
Never measured	73.4 (70.1–76.5)	31.5 (28.7–34.4)	49.2 (46.9–51.6)
Hypertensive	9.7 (7.8–12.1)	12.4 (10.5–14.6)	11.3 (9.9–12.9)
Non‐hypertensive	16.8 (14.3–19.7)	56.1 (53.0–59.1)	39.5 (37.2–42.8)
History of raised cholesterol			
Never tested	97.1 (95.6–98.1)	91.1 (89.1–92.7)	93.6 (92.4–94.7)
High cholesterol	1.8 (1.0–3.0)	1.5 (0.9–2.5)	1.6 (1.1–2.3)
Normal cholesterol	1.1 (0.5–2.1)	7.4 (5.9–9.2)	4.7 (3.8–5.9)
History of heart attack or chest pain	6.6 (5.0–8.6)	8.1 (6.6–10.0)	7.5 (6.3–8.8)

Abbreviation: CI, confidence interval.

### 3.5 Physical Measurements

The prevalence of high BP (SBP ≥ 140 mmHg and/or DBP ≥ 90 mmHg) was 27.8%. Nigerian participants had an average weight of 72.2 kg, while Ethiopians had 60.4 kg. The overall mean body mass index (BMI) was 26.5 kg/m^2^, with Nigerians having a higher BMI (28.6 kg/m^2^) than Ethiopians (23.6 kg/m^2^). Nearly two‐thirds of the surveyed participants (63.5%) had normal metabolic risk based on waist circumference as shown in Table 5.

**TABLE 5:** Distribution of raised BP, weight, height, BMI, waist circumference, and WHR by country of residence, Ethiopia and Nigeria, 2021.

Variables	Ethiopia	Nigeria	Total
Blood pressure			
Raised BP, % (95% CI)	17.7 (15.1–20.6)	35.6 (32.5–38.5)	27.8 (25.8–30.0)
Raised SBP, % (95% CI)	11.1 (9.0–13.6)	31.1 (28.3–34.1)	22.6 (20.6–24.6)
Raised DBP, % (95% CI)	14.1 (11.8–16.8)	25.0 (22.4–27.8)	20.3 (18.5–22.3)
Weight and height^a^			
Mean weight, kg (95% CI)	60.4 (59.6–61.3)	72.2 (71.2–73.3)	67.1 (66.4–67.9)
Mean height, cm (95% CI)	159.9 (159.3–160.6)	161.5 (160.6–162.3)	160.8 (160.2–161.4)
BMI^b^, ^c^ % (95% CI)			
Mean BMI	23.6 (23.3–24.0)	28.6 (27.6–29.6)	26.5 (25.9–27.1)
Underweight	7.0 (5.4–9.1)	2.4 (1.6–3.5)	4.4 (3.5–5.4)
Normal	63.3 (59.7–66.7)	37.8 (34.8–40.9)	48.8 (46.4–51.1)
Overweight	21.2 (18.4–24.4)	29.7 (26.9–32.6)	26.0 (23.0–28.2)
Obese	8.5 (6.7–10.7)	30.2 (27.4–33.1)	20.9 (19.0–22.8)
Waist circumference^d^, % (95% CI)			
Normal risk	80.6 (77.5–83.3)	50.8 (47.6–53.9)	63.5 (61.2–65.8)
Increased risk	11.1 (9.0–13.6)	20.7 (18.3–23.3)	16.6 (14.9–18.4)
Substantially increased risk	8.4 (6.6–10.6)	28.6 (25.8–31.5)	19.9 (18.1–21.9)
Waist to hip ratio (WHR), % (95% CI)			
Mean WHR	0.85 (0.84–0.86)	NA	0.85 (0.84–0.86)
Normal	65.0 (61.5–68.4)	NA	65.0 (61.5–68.4)
Obese	35.0 (31.6–38.5)	NA	35.0 (31.6–38.5)

Abbreviations: BP, blood pressure; BMI, body mass index; CI, confidence interval; DBP, diastolic blood pressure; SBP, systolic blood pressure; WHR, waist‐to‐hip ratio.

^a^ Overall, 27 values were missing.

^b^ Overall, 29 values were missing.

^c^ WHO cut off for BMI classification is used.

^d^ Waist circumference classification is based on WHO recommendation where normal risk is ≤ 94 cm for men and ≤ 80 cm for women; increased risk is > 94–102 cm for men and > 80–88 cm for women; and substantial increased risk is > 102 cm for men and > 88 cm for women.

### 3.6 Prevalence of Diabetes and Prediabetes

In the survey, 15.8% (265 individuals) met prediabetes criteria. Impaired fasting glycaemia (IFG) was present in 11.8%, while 11.5% exhibited impaired glucose tolerance (IGT). Among 1550 individuals tested by both fasting and OGTT, isolated IFG and IGT were observed in 5.5% and 5.8% of participants, respectively (Table 6).

**TABLE 6:** Percentage of prediabetes and diabetes by sex and country of residence, Ethiopia and Nigeria, 2021.

Test results	Number of tests	Sex		Country		Total, *n* (%)
		Men, *n* (%)	Women, *n* (%)	Ethiopia, *n* (%)	Nigeria, *n* (%)	
Prediabetes						
Impaired fasting glucose	1614	101 (12.4)	89 (11.2)	31 (4.8)	159 (16.4)	190 (11.8)
Impaired glucose tolerance	1588	105 (13.3)	79 (9.9)	27 (4.1)	156 (16.8)	184 (11.5)
Isolated impaired fasting glucose	1550	45 (5.8)	38 (4.9)	13 (2.1)	70 (7.5)	83 (5.4)
Isolated impaired glucose tolerance	1550	53 (6.8)	39 (5.1)	15 (2.4)	77 (8.3)	92 (5.9)
All prediabetes	1682	146 (17.2)	119 (14.3)	41 (5.8)	224 (22.9)	265 (15.8)
Diabetes						
Known people with diabetes	1725	20 (2.3)	10 (1.2)	19 (2.6)	11 (1.1)	30 (1.7)
New diabetes by fasting	1614	42 (5.1)	12 (1.5)	4 (0.6)	50 (5.2)	54 (3.3)
New diabetes by 2 h OGTT	1588	35 (4.4)	28 (3.5)	20 (3.0)	43 (4.6)	63 (4.0)
All newly diagnosed diabetes	1574	58 (7.3)	30 (3.9)	20 (3.2)	68 (7.2)	88 (5.6)
Overall prevalence of diabetes	1682	78 (9.2)	40 (4.8)	39 (5.5)	79 (8.1)	118 (7.0)

Abbreviation: 2 h OGTT, 2‐hour oral glucose tolerance test.

**FIGURE 1:**
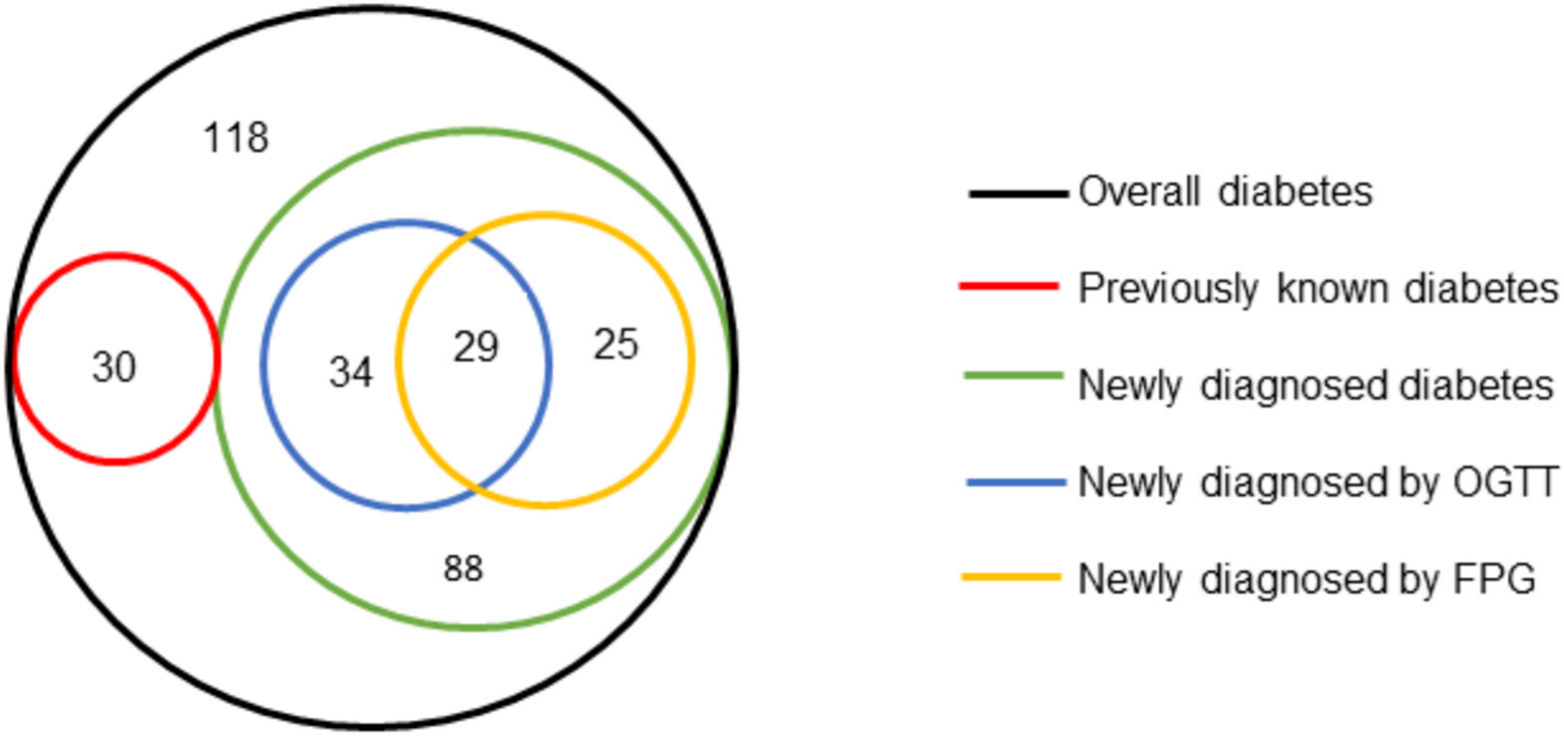
Number of previously diagnosed and newly diagnosed diabetes cases identified by FBG and 2 h OGTT, Ethiopia and Nigeria, 2021. FPG, fasting plasma glucose; OGTT, oral glucose tolerance test.

Among the survey participants, 118 individuals (7.0%) met at least one of the criteria for diabetes. Three‐fourths (74.6%) of the people diagnosed with diabetes were not aware of their diabetes status prior to the survey, yielding a 5.6% prevalence of undiagnosed diabetes in the region. In addition to 30 (1.7%) participants with a history of diabetes, we identified 54 (3.4%) new individuals with diabetes by FPG measurement and 63 (4.0%) by OGTT (Figure 1).

As presented in Table 7, the mean (SD) age of Ethiopians with diabetes was 47.4 years (SD 14.2), nearly 7 years older compared to the non‐diabetic cases. Furthermore, the mean weight among Ethiopians with diabetes is 68.6 kg (SD 17.0 kg), significantly higher than their non‐diabetic counterparts (*p* < 0.001). Similarly, Ethiopians with diabetes had significantly higher mean BMI (26.8 ± 5.8 kg/m^2^) and waist circumference (85.1 ± 13.7 cm) compared to non‐diabetic participants (*p* < 0.01).

**TABLE 7:** Characteristics of study participants by diabetes status and country, Ethiopia and Nigeria (*N* = 1682).

Variables	Total (*N* = 1682)	Ethiopia (*n* = 704)				Nigeria (*n* = 978)			
		Normal (*n* = 625)	Prediabetes (*n* = 41)	Diabetes (*n* = 39)	*p*	Normal (*n* = 678)	Prediabetes (*n* = 224)	Diabetes (*n* = 79)	*p*
Men	848 (50.4)	226 (36.2)	14 (34.1)	18 (46.2)	0.432	398 (59.0)	132 (58.9)	60 (75.9)	0.013
Age (years)	1682	40.5 ± 13.5	42.5 ± 13.1	47.4 ± 14.2	0.007	45.2 ± 18.2	50.7 ± 15.9	53.5 ± 14.2	< 0.001
Urban	883 (52.5)	248 (39.7)	15 (36.6)	9 (23.1)	0.112	346 (51.3)	122 (54.5)	59 (74.7)	< 0.001
Current smoker	114 (6.8)	33 (5.3)	—	1 (2.6)	0.246	56 (8.3)	20 (8.9)	4 (5.1)	0.549
Current alcohol use	468 (27.8)	94 (15.1)	8 (19.5)	3 (7.7)	0.317	241 (35.7)	90 (40.2)	32 (40.5)	0.393
Current Khat chewer	414 (58.8)	372 (59.6)	20 (48.8)	22 (56.4)	0.375	NA	NA	NA	NA
Number of days fruits consumed in a week	1682	1.7 ± 1.12	1.9 ± 1.10	1.5 ± 1.04	0.299	2.9 ± 1.90	3.2 ± 2.10	2.6 ± 1.98	0.073
Consumed fruits in the past week	1460 (86.8)	564 (90.4)	39 (95.1)	34 (87.2)	0.705	571 (84.6)	192 (85.7)	60 (75.9)	0.075
Number of days vegetables consumed in a week	1682	4.2 ± 2.19	3.9 ± 2.16	3.9 ± 1.76	0.518	4.2 ± 2.30	4.5 ± 2.17	3.5 ± 2.41	0.005
Consumed vegetables in the past week	1521 (90.4)	603 (96.6)	39 (95.1)	39 (100.0)	0.652	578 (85.6)	202 (90.2)	60 (75.9)	0.031
Optimal physical activity^a^	1317 (78.3)	478 (76.6)	32 (78.0)	29 (74.4)	0.925	535 (79.3)	183 (81.7)	60 (75.9)	0.522
Weight (kg)	1674	59.5 ± 10.8	65.0 ± 14.3	68.6 ± 17.0	< 0.001	70.2 ± 14.6	74.3 ± 17.0	83.9 ± 25.5	< 0.001
BMI	1674	23.3 ± 4.1	25.9 ± 5.4	26.8 ± 5.8	< 0.001	27.7 ± 17.9	29.0 ± 11.9	35.5 ± 12.6	< 0.001
Obese	351 (21.0)	39 (6.3)	8 (15.8)	12 (30.8)	< 0.001	159 (23.7)	83 (37.6)	50 (64.9)	< 0.001
WC, overall	1682	76.3 ± 10.7	81.4 ± 11.5	85.1 ± 13.7	< 0.001	87.4 ± 13.4	91.1 ± 14.8	95.4 ± 15.1	< 0.001
WC, men	848	78.4 ± 8.9	85.4 ± 9.5	87.1 ± 14.9	< 0.001	88.2 ± 13.9	91.8 ± 16.7	96.6 ± 15.9	< 0.001
WC, women	834	75.1 ± 11.4	79.2 ± 12.0	83.4 ± 12.8	0.002	86.2 ± 12.6	90.0 ± 11.6	91.8 ± 11.9	0.012
Substantially increased risk of central obesity	337 (20.0)	39 (6.3)	8 (19.5)	11 (28.2)	< 0.001	166 (24.6)	80 (35.7)	33 (42.3)	< 0.001
WHR	703	0.85 ± 0.12	0.87 ± 0.10	0.88 ± 0.11	0.242	NA	NA	NA	NA
Abnormal WHR	251 (35.7)	210 (33.7)	17 (41.5)	24 (63.2)	< 0.001	NA	NA	NA	NA
DBP (mm Hg)	1680	78.4 ± 10.7	83.7 ± 14.2	82.1 ± 12.2	0.002	80.5 ± 12.5	85.4 ± 13.9	83.7 ± 12.3	< 0.001
SBP (mm Hg)	1680	118.3 ± 16.3	129.2 ± 19.4	124.3 ± 18.4	< 0.001	130.1 ± 20.7	136.7 ± 23.0	140.0 ± 23.3	< 0.001
Raised BP	470 (28.0)	100 (16.0)	10 (24.4)	14 (35.9)	0.003	206 (30.6)	98 (43.8)	42 (53.8)	< 0.001
Reported history of hypertension	193 (11.5)	51 (8.2)	4 (9.8)	14 (35.9)	< 0.001	60 (8.9)	44 (19.6)	20 (25.3)	< 0.001
Reported history of high cholesterol	28 (1.7)	7 (1.1)	1 (2.4)	5 (12.8)	0.005	8 (1.2)	6 (2.7)	1 (1.3)	0.255

*Note:* Data are presented as *n* (%), mean ± SD.

Abbreviations: BMI, body mass index; NA, not applicable; SD, standard deviation; WHO, World Health Organization; WHR, waist‐to‐hip ratio.

^a^ WHO global recommendations on physical activity.

The mean (SD) age among Nigerians with diabetes was 53.5 years (SD 14.2), which was over 8 years higher than the mean age of non‐diabetic cases, recorded at 45.2 years (SD 18.2). Moreover, Nigerians with diabetes had a higher mean weight of 83.9 kg (SD 25.5 kg) compared to their non‐diabetic counterparts (*p* < 0.001). In addition, Nigerian diabetes cases had significantly higher mean BMI, waist circumference, DBP, and SBP as compared to non‐diabetic participants (*p* < 0.001).

With the multivariable logistic regression analysis (Data S1), sex is identified as a common predictor of the prevalence of Type 2 diabetes in both Nigeria and Ethiopia. Ethiopian males were nearly four times more likely to have DM than their female counterparts (AOR = 3.80, 95% CI = 1.22–11.82, *p* = 0.021), while Nigerian males were nearly three times more likely to have DM than females (AOR 2.89, 95% CI 1.35–6.18, *p* 0.007). In addition, older age and urban residence are significant predictors in Nigeria, with urban dwellers over twice as likely to develop type 2 diabetes (AOR = 2.45, 95% CI = 1.33–4.49, *p* = 0.004). Central obesity is also a strong predictor in Ethiopia, with higher risks linked to greater odds of DM; whereas, weight is a significant factor instead in Nigeria. Hypertension is also a key predictor in Ethiopia, increasing DM odds by 4% (AOR 3.68, 95% CI 1.07–12.61, *p* 0.038).

## 4 Discussion

The aim of this study is to explore the prevalence and phenotypes of Type 2 diabetes among Ethiopian and Nigerian participants, the two most populous countries in Africa. The findings from this study are based on data from Butajira in Ethiopia and Enugu State in Nigeria and should not be generalized to all of SSA without further research. We found an overall prevalence of diabetes to be 7.0%; higher among Nigerians (8.1%) than the Ethiopians (5.5%).

According to WHO STEPS reports [7], the prevalence of Type 2 diabetes in SSA ranged from 1.4% to 22.5%. The prevalence of Type 2 diabetes in the current study (7.0%) is higher than findings reported from Zambia (3.5%) [12] and Kenya (4.4%) [13] but lower than a study in South Africa (15.3%) [14]. The prevalence of diabetes in Butajira, Ethiopia 5.5% corresponds to the national prevalence estimate of 5.0% by IDF in 2021 [2], but exceeds the 2015 National NCDs STEPS Survey (3.2%) [15]. The 8.1% prevalence among Nigerian participants contrasts with a previous systematic review and meta‐analysis showing a pooled prevalence of diabetes at 5.7% [16]. The discrepancies in these findings may stem from differences in diagnostic criteria and tests, and sampling methods; limiting direct comparison. Unlike earlier studies relying mainly on random or FPG, this research employed both OGTT and FPG to identify diabetes cases.

Alarmingly, three‐quarters (74.6%) of the individuals with diabetes were not aware of their diabetes status prior to the survey, comparable with the previous findings in Nairobi (75.0%) [13]. Failure to diagnose diabetes promptly and, if left untreated, it can lead to severe complications, enduring disabilities, and premature mortality.

This study identified a prediabetes prevalence of 15.8%, comparable to rates in previous Ethiopian [17] and Nigerian [18] studies, suggesting an increased risk of progression of prediabetes to diabetes. Among prediabetes cases, 11.8% had IFG, whereas 11.5% had IGT. Among the study participants tested by both OGTT and fasting, isolated‐IFG was found in 5.4% of participants, while isolated‐IGT was present in 5.9% of participants. The slightly higher I‐IGT rates emphasize the importance of OGTTs for early detection of abnormal glucose regulation, better identifying those at risk for diabetes and its complications. In individuals with I‐IFG, the major disturbance is hepatic insulin resistance, while I‐IGT individuals have normal to slightly reduced hepatic insulin sensitivity and moderate‐to‐severe muscle insulin resistance; those with combined IFG and IGT tend to have both muscle and hepatic insulin resistance [19].

The African continent has the most genetic heterogeneity in the world that could implicate the diversified clinical profiles of diabetes [20]. SSA is also unique in that it continues to have a high rate of infectious diseases and other problems including famine, civil conflict, and malnutrition, which may modulate the pathogenesis and clinical course of diabetes. Famine and food insecurity related history of Ethiopia may have led to metabolic changes that could result in variations in different phenotypes in insulin resistance and diabetes [21, 22]. In Nigeria, the history of malnutrition, particularly in civil‐conflict affected areas, could have led to insufficient dietary consumptions and contributed to diversified diabetes phenotypic characteristics including differences in obesity levels, dyslipidemia, and other chronic diseases [23].

The study found that adults diagnosed with diabetes in Ethiopia are younger, indicating early disease onset, comparable to a study in Indian [24]. Prediabetes was the highest in the 45–59 age group in Ethiopia, whereas it was invariably higher after age 30 in Nigeria. Ethiopian participants with diabetes had lower body weight, waist circumference, and WHR, comparable to previous studies in Ethiopia [25] and Asian [26, 27]. In contrast, diabetes in Western individuals appears to develop later in life (> 50 years) and is common among overweight and obese individuals [28]. In line with this, multivariable analysis showed that age was significantly associated with type 2 diabetes in Nigerian individuals. Unlike the Ethiopian participants, Nigerians showed phenotypes more comparable to Western individuals.

Central obesity is found to be a strong predictor of DM in Ethiopia, where individuals with increased risk of central obesity are more likely to have Type 2 diabetes compared to low risk for central obesity as measured by waist circumference. These findings highlight the critical role of abdominal fat in driving Type 2 diabetes prevalence in Ethiopia. In contrast, central obesity is not found to be a predictor of Type 2 diabetes in Nigeria, but higher weight is significantly associated with Type 2 diabetes. This suggests that waist cut‐offs for waist circumference for predicting Type 2 diabetes in African populations vary significantly from international standards, highlighting the need for tailored thresholds [29, 30].

In overweight or obese individuals, Type 2 diabetes is most likely to result from adiposity‐induced insulin resistance, followed by compensatory insulin secretion and subsequent β‐cell dysfunction, and ultimately manifest with overt hyperglycemia [31]. Conversely, in individuals who are not overweight, the underlying pathophysiology is not well comprehended and is often ascribed to the deposition of ectopic fat [32] and impairments in insulin secretion [33]. Primary pancreatic beta cell secretory dysfunction might explain why diabetes in Ethiopia is occurring at a lower age, BMI, waist circumference, and waist‐to‐hip ratio, while insulin resistance could be important among Nigerian participants, as the majority (64.9%) of them were obese. In line with this, a previous Nigerian study reported a high dual burden of insulin resistance and pancreatic beta cell secretory dysfunction among individuals with Type 2 diabetes [34].

Hypertension often coexists with Type 2 diabetes [35]. In the present study, it was also observed that a significant proportion of individuals with diabetes had associated hypertension (53.8% among the Nigerians and 35.9% for Ethiopians) compared to non‐diabetic cases (30.6% among Nigerians and 16.0% among Ethiopians). In line with this, Ethiopian individuals reporting a history of hypertension were more likely to have Type 2 diabetes than individuals without a history of hypertension. This dual adversity raises cardiovascular risk, demanding strict control of blood pressure and blood sugar levels. Hypertension also worsens micro‐ and macrovascular complications in diabetes, stressing the need for effective management strategies.

In SSA, the clinical management of adults with diabetes is predominantly guided by clinical guidelines formulated by international diabetes associations such as ADA [4]. It is noteworthy, however, that these international guidelines are developed from evidence generated through phenotyping studies conducted on Caucasian or mixed ancestry populations. Hence, it might not be applicable directly to the SSA population.

## 5 Conclusion

In the present study, there is a notable occurrence of diabetes at a relatively high rate of 7.0% among rural and peri‐urban populations in SSA, higher among Nigerians than Ethiopians. The prevalence of prediabetes was 16.5% in the present study. Given that three‐quarters of diabetes cases were undiagnosed, there may be a large number of people with diabetes in the study settings who do not know their status. It is recommended that screening and early detection efforts be intensified in SSA.

The findings of this study showed that individuals with diabetes in Ethiopia compared to Nigeria are characterized by a young age of onset, leanness, lower BMI, and waist circumference. The diabetes phenotype encountered in Ethiopia is very different from the classical Type 2 diabetes seen in the West, while Nigerian diabetes cases had comparable phenotypes to Westerners. This underscores the imperative need for further research and careful consideration when formulating therapeutic strategies for diabetes specifically tailored to the distinct physiological and clinical characteristics of populations in SSA.

This research relied on data from specific study areas within Ethiopia (Butajira) and Nigeria (Enugu State). While the findings from these areas provide important insights on the burden and clinical profiles of Type 2 diabetes, it is important to emphasize that the results may not be generalized to all of SSA. Because of genetic and environmental variabilities across SSA, the prevalence of Type 2 diabetes may vary considerably across the region. Nevertheless, the inclusion of individuals from Ethiopia and Nigeria, which together account for a substantial proportion of SSA's population, offers important insight that can inform future studies and public health strategies. Further studies on a wider range of countries from the region are recommended to build a more comprehensive understanding of the epidemiology of diabetes in SSA.

## 6 Strength and Limitation

This study employed a community‐based multi‐center design, facilitating the extrapolation of outcomes to the broader population of the research settings within SSA. In contrast to numerous prior investigations in the region that relied on random or fasting blood glucose measurements, we have used both fasting and 2‐h OGTT, thereby enhancing the accuracy of diabetes cases. However, it is crucial to acknowledge that the cross‐sectional nature of the design precludes the confirmation of the course of causal pathways.

## Author Contributions

Assefa Mulu Baye, Adem Y. Dawed, Teferi Gedif Fenta, Ewan R. Pearson, and Colin Palmer designed the study. Assefa Mulu Baye, Ifeyinwa Dorothy Nnakenyi, Ekenechukwu Esther Young, and Ifeoma Isabella Ulasi conducted the research. Assefa Mulu Baye, Ifeyinma Dorothy Nnakeyni, and Teferi Gedif Fenta contributed to the data analysis and interpretation. Assefa Mulu Baye, Adem Y. Dawed, and Ifeoma Isabella Ulasi performed statistical analysis. Assefa Mulu Baye and Adem Y. Dawed wrote the draft manuscript. All the authors contributed to the discussion and revision of the manuscript. Assefa Mulu Baye confirmed that all authors included in the list meet the necessary authorship criteria. As the guarantor of this work, Assefa Mulu Baye had complete access to all data analyzed in the study and bears responsibility for ensuring the data's integrity and accuracy in the analysis.

## Ethics Statement

This study was conducted with the approval of the Institutional Review Board of the College of Health Sciences, Addis Ababa University (Ref: 027/19/SoP), Ministry of Education, Ethiopia (Ref: 17/152/407/23) and University of Nigeria Teaching Hospital, Enugu, Health Research Ethics Committee (Ref: UNTH/CSA/329/OL.5). Written informed consent was obtained from the participants.

## Conflicts of Interest

The authors declare no conflicts of interest.

## Supporting information


**Data S1.** Supporting Information.
