# Supplementary material for: Comparative Analysis of Type 2 Diabetes Prevalence and Clinical Profiles in Ethiopia and Nigeria
Source: J Diabetes. 2025 Apr 2;17(4):e70078. doi: 10.1111/1753-0407.70078 (PMC11962519; doi:10.1111/1753-0407.70078)
Supplement: Supplementary file 1 — Data S1. Supporting Information. [file JDB-17-e70078-s001.docx]

**Supplementary materials**

Characteristics of cases by the refusal of blood tests

| **Characteristics** | **Test status** | | **p-value** |
| --- | --- | --- | --- |
|  | **Refused for blood tests** | **Provide blood tests** |  |
| Sex |  |  |  |
| Male | 122 (45.2) | 872 (50.5) | 0.105 |
| Female | 148 (54.8) | 855 (49.5) |  |
| Age, mean (SD) | 43.4 (9.2) | 44.6 (16.3) | 0.134 |
| Residence |  |  |  |
| Urban | 149 (55.2) | 918 (53.2) | 0.534 |
| Rural | 121 (44.8) | 809 (46.8) |  |
| Educational status |  |  |  |
| No formal education | 87 (24.8) | 461 (26.7) | 0.191 |
| Some primary education | 57 (21.1) | 265 (15.3) |  |
| Completed primary education | 30 (11.1) | 227 (13.1) |  |
| Junior secondary education | 18 (6.7) | 94 (5.4) |  |
| Senior secondary education | 44 (16.3) | 283 (16.4) |  |
| Above secondary education | 54 (20.0) | 397 (23.0) |  |
| Current cigarette smoking |  |  |  |
| Yes | 16 (5.9) | 116 (6.7) | 0.627 |
| No | 254 (94.1) | 161 (93.3) |  |
| Current alcohol drinking |  |  |  |
| Yes | 61 (22.6) | 474 (27.4) | 0.094 |
| No | 209 (77.4) | 1253 (72.6) |  |
| Number of days fruits consumed in a week, mean (SD) | 2.5 (1.1) | 2.4 (1.8) | 0.364 |
| Number of servings of fruits per day, mean (SD) | 1.2 (0.5) | 1.3 (0.6) | 0.402 |
| Number of days vegetables consumed per a week, mean (SD) | 4.4 (1.4) | 4.2 (2.2) | 0.112 |
| Number of servings of vegetables per day, mean (SD) | 1.8 (0.6) | 1.9 (0.9) | 0.185 |
| Weight in kg, mean (SD) | 65.8 (9.1) | 67.1 (15.9) | 0.094 |
| BMI in kg/m2, mean (SD) | 25.4 (2.9) | 26.5 (12.9) | 0.189 |
| History of hypertension |  |  |  |
| No checkup | 138 (51.1) | 850 (49.2) | 0.744 |
| Yes | 32 (11.9) | 195 (11.3) |  |
| No | 100 (37.0) | 682 (39.5) |  |

Abbreviations: SD, standard deviation

Comparison of Predictors of Type 2 Diabetes Prevalence in Ethiopia and Nigeria:

Ethiopia

| **Characteristics** | **Diabetes Mellitus** | | **COR (95% CI)** | **P-value** | **AOR (95% CI)** | **P-value** |
| --- | --- | --- | --- | --- | --- | --- |
|  | **Non-diabetes** | **Diabetes** |  |  |  |  |
| **Sex** | | | | | | |
| Male | 240 (93.0) | 18 (7.0) | 1.52 (0.79-2.91) | 0.208 | 3.80 (1.22-11.82) | **0.021** |
| Female | 425 (95.3) | 21 (4.7) | 1.00 |  | 1.00 |  |
| **Age** |  |  |  |  |  |  |
| Mean (SD) | 40.6 (13.5) | 47.4 (14.2) | 1.03 (1.01-1.06) | **0.003** | 1.04 (0.94-1.16) | 0.438 |
| 18-29 years | 139 (97.2) | 4 (2.8) | 1.00 |  | 1.00 |  |
| 30-44 years | 286 (95.3) | 14 (4.7) | 1.70 (0.55-5.26) | 0.357 | 0.55 (0.10-3.03) | 0.488 |
| 45-59 years | 153 (93.3) | 11 (6.7) | 2.50 (0.78-8.03) | 0.124 | 0.24 (0.01-4.69) | 0.343 |
| 60-69 years | 58 (89.2) | 7 (10.8) | 4.19 (1.18-14.88) | **0.026** | 0.69 (0.01-49.18) | 0.862 |
| ≥70 years | 29 (90.60 | 3 (9.4) | 3.60 (0.76-16.93) | 0.106 | 0.20 (0.00-53.32) | 0.571 |
| **Residence** |  |  |  |  |  |  |
| Urban | 402 (93.1) | 30 (6.9) | 2.18 (1.02-4.67) | **0.045** | 2.00 (0.54-7.28) | 0.301 |
| Rural | 263 (96.7) | 9 (3.3) | 1.00 |  | 1.00 |  |
| **Current smoker** |  |  |  |  |  |  |
| Yes | 33 (97.1) | 1 (2.9) | 0.50 (0.07-3.79) | 0.505 | 0.29 (0.03-2.66) | 0.271 |
| No | 632 (94.3) | 38 (5.7) | 1.00 |  | 1.00 |  |
| **Current alcohol drinker** |  |  |  |  |  |  |
| Yes | 102 (97.1) | 3 (2.9) | 0.46 (0.14-1.52) | 0.203 | 0.68 (0.18-2.59) | 0.574 |
| No | 563 (94.0) | 36 (6.0) | 1.00 |  | 1.00 |  |
| **Dietary factors** |  |  |  |  |  |  |
| No. of days fruits consumed in a week, mean (SD) | 1.7 (1.1) | 1.5 (1.0) | 0.83 (0.60-1.15) | 0.268 | 0.80 (0.50-1.26) | 0.332 |
| Consumed fruits in the past week |  |  |  |  |  |  |
| No | 50 (94.3) | 3 (5.7) | 1.03 (0.31-3.45) | 0.968 | 0.91 (0.18-4.53) | 0.907 |
| Yes | 615 (94.5) | 36 (5.5) | 1.00 |  | 1.00 |  |
| No. of days vegetables consumed in a week | 4.1 (2.2) | 3.9 (1.8) | 0.83 (0.60-1.15) | 0.268 | 1.14 (0.88-1.48) | 0.321 |
| **Optimal physical activity^a^** |  |  |  |  |  |  |
| Suboptimal | 155 (93.9) | 10 (6.1) | 1.14 90.54-2.38) | 0.738 | 1.11 (0.45-3.02) | 0.746 |
| Optimal | 510 (94.6) | 29 (5.4) | 1.00 |  | 1.00 |  |
| **Anthropometries** |  |  |  |  |  |  |
| Weight in kg, mean (SD) | 59.9 (11.2) | 68.6 (17.1) | 1.06 (1.03-1.08) | **<0.001** | 1.00 (0.93-1.06) | 0.881 |
| BMI in kg/m2, mean (SD) | 23.4 (4.2) | 26.8 (16.6) | 1.14 (1.08-1.21) | **<0.001** | 0.97 90.79-1.21) | 0.813 |
| WC in cm, mean (SD) | 76.6 (10.8) | 85.1 (13.7) | 1.05 (1.03-1.08) | **<0.001** | 0.97 (0.89-1.05) | 0.431 |
| **Risk of central obesity** |  |  |  |  |  |  |
| Low risk | 546 (96.5) | 20 (3.5) | 1.00 |  | 1.00 |  |
| Increased riisk | 72 (90.0) | 8 (10.0) | 3.03 (1.29-7.14) | **0.011** | 4.55 (1.19-17.43) | **0.027** |
| Substantally increased risk | 47 (81.0) | 11 (19.0) | 6.39 (2.90-14.13) | **<0.001** | 8.84 (1.31-59.59) | **0.025** |
| **Blood pressure, mmHg** |  |  |  |  |  |  |
| DBP | 78.7 (11.0) | 82.1 (12.2) | 1.03 (1.00-1.05) | 0.067 | 1.01 (0.95-1.07) | 0.829 |
| SBP | 119.0 (16.7) | 124.3 (18.4) | 1.02 (1.00-1.04) | 0.055 | 0.98 (0.94-1.02) | 0.262 |
| **Raised BP** |  |  |  |  |  |  |
| Normal | 555 (95.7) | 25 (4.3) | 1.00 |  | 1.00 |  |
| Raised | 110 (88.7) | 114 (11.3) | 2.83 (1.42-5.61) | 0.003 | 1.97 (0.51-7.69) | 0.327 |
| **Reported history of hypertension** |  |  |  |  |  |  |
| No checkup | 501 (96.5) | 18 (3.5) | 0.56 (0.23-1.37) | 0.205 | 0.86 (0.29-2.51) | 0.779 |
| Yes | 55 (79.7) | 14 (20.3) | 3.96 (1.51-10.39) | **0.005** | 3.68 (1.07-12.61) | **0.038** |
| No | 109 (94.0) | 7 (6.0) | 1.00 |  | 1.00 |  |
| **Reported history of high cholesterol** |  |  |  |  |  |  |
| No checkup | 650 (95.2) | 33 (4.8) | 0.36 (0.04-2.97) | 0.340 | 0.75 (0.04-12.72) | 0.839 |
| Yes | 8 (61.5) | 5 (38.5) | 4.38 (0.41-47.02) | 0.223 | 3.80 (0.17-83.00) | 0.396 |
| No | 7 (87.5) | 1 (12.5) | 1.00 |  | 1.00 |  |

Abbreviations: AOR, adjusted odds ratio; CI, confidence interval; COR, crude odds ratio; DBP, diastolic blood pressure; SBP; systolic blood pressure; SD, standard deviation

^a^ WHO global recommendations on physical activity

Nigeria

| **Characteristics** | **Diabetes Mellitus** | | **COR (95% CI)** | **P-value** | **AOR (95% CI)** | **P-value** |
| --- | --- | --- | --- | --- | --- | --- |
|  | **Non-diabetes** | **Diabetes** |  |  |  |  |
| Sex | | | | | | |
| Male | 530 (89.8) | 60 (10.2) | 2.20 (1.29-3.75) | **0.004** | 2.89 (1.35-6.18) | **0.007** |
| Female | 369 (95.1) | 19 (4.9) | 1.00 |  | 1.00 |  |
| Age |  |  |  |  |  |  |
| Mean (SD) | 46.6 (17.8) | 53.5 (14.2) | 1.02 (1.01-1.04) | **<0.001** | 0.97 (0.91-1.03) | 0.293 |
| 18-29 years | 183 (97.9) | 4 (2.1) | 1.00 |  | 1.00 |  |
| 30-44 years | 268 (94.7) | 15 (5.3) | 2.56 (0.84-7.84) | **0.100** | 3.06 (0.72-12.91) | 0.129 |
| 45-59 years | 188 (86.2) | 30 (13.8) | 7.30 (2.52-21.13) | **<0.001** | 12.56 (1.60-98.47) | **0.016** |
| 60-69 years | 132 (88.6) | 17 (11.4) | 5.89 (1.94-17.91) | **0.002** | 14.80 (0.95-231.74 | 0.055 |
| ≥70 years | 128 (90.8) | 13 (9.2) | 4.65 (1.48-14.58) | **0.008** | 21.54 (0.70-659.87) | 0.079 |
| **Residence** |  |  |  |  |  |  |
| Urban | 468 (88.8) | 59 (11.2) | 2.7 (1.61-4.45 | **<0.001** | 2.45 (1.33-4.49) | **0.004** |
| Rural | 431 (95.6) | 20 (4.4) | 1.00 |  | 1.00 |  |
| **Current smoker** |  |  |  |  |  |  |
| Yes | 76 (95.0) | 4 (5.0) | 0.58 (0.21-1.62) | 0.298 | 1.18 (0.35-3.97) | 0.789 |
| No | 823 (91.6) | 75 (8.4) | 1.00 |  | 1.00 |  |
| **Current alcohol drinker** |  |  |  |  |  |  |
| Yes | 331 (91.2) | 32 (8.8) | 1.17 (0.73-1.87) | 0.516 | 1.62 (0.92-3.840 | 0.094 |
| No | 568 (92.4) | 47 (7.6) | 1.00 |  | 1.00 |  |
| **Dietary factors** |  |  |  |  |  |  |
| No**. of days fruits consumed in a week** | 3.0 (1.9) | 2.9 (1.9) | 0.89 (0.79-1.01) | 0.071 | 0.94 (0.79-1.12) | 0.473 |
| Consumed **fruits in the past week** |  |  |  |  |  |  |
| No | 111 (87.4) | 16 (12.6) | 1.80 (1.01-3.23) | **0.048** | 0.68 (0.23-1.95) | 0.409 |
| Yes | 788 (92.6) | 63 (7.4) | 1.00 |  | 1.00 |  |
| **No. of days vegetables consumed in a week** | 4.3 (2.3) | 3.5 (2.4) | 0.89 (0.79-1.01) | 0.071 | 0.87 (0.75-1.01) | 0.073 |
| **Optimal physical activity** |  |  |  |  |  |  |
| Suboptimal | 181 (90.5) | 19 (9.5) | 1.26 (0.73-2.16) | 0.409 | 1.17 (0.64-2.17) | 0.608 |
| Optimal | 718 (92.3) | 60 (7.7) | 1.00 |  | 1.00 |  |
| **Anthropometries** |  |  |  |  |  |  |
| Weight | 71.2 (15.4) | 83.9 (25.5) | 1.04 (1.03-1.05) | **<0.001** | 1.04 (1.02-1.06) | **<0.001** |
| BMI | 28.0 (16.6) | 35.5 (12.6) | 1.02 (1.00-1.03) | **0.034** | 0.99 (0.98-1.01) | 0.593 |
| Waist circumferemce | 88.3 (13.8) | 95.4 (15.1) | 1.03 (1.02-1.05) | **<0.001** | 0.99 (0.96-1.03) | 0.689 |
| Risk of central obesity |  |  |  |  |  |  |
| Low risk | 469 (94.6) | 27 (5.4) | 1.00 |  | 1.00 |  |
| Increased riisk | 184 (91.1) | 18 (8.9) | 1.70 (0.91-3.16) | 0.094 | 1.40 (0.60-3.29) | 0.438 |
| Substantally increased risk | 246 (88.2) | 33 (11.8) | 2.33 (1.37-3.97) | **0.002** | 1.85 (0.63-5.43) | 0.266 |
| **DBP** | 81.7 (13.0) | 83.7 (12.3) | 1.01 (0.99-1.03) | 0.208 | 0.97 (0.95-1.00) | 0.057 |
| **SBP** | 131.7 (21.5) | 140.0 (23.3) | 1.02 (1.01-1.03) | **0.001** | 1.01 (0.99-1.03) | 0.305 |
| **Raised BP** |  |  |  |  |  |  |
| Normal | 594 (94.3) | 36 (5.7) | 1.00 |  | 1.00 |  |
| Raised | 304 (87.9) | 42 (12.1) | 2.28 (1.43-3.63) | **<0.001** | 1.44 (0.60-3.42) | 0.413 |
| **Reported history of hypertension** |  |  |  |  |  |  |
| No checkup | 289 (94.8) | 16 (5.2) | 0.65 (0.36-1.18) | 0.156 | 0.77 (0.40-1.50) | 0.449 |
| Yes | 104 (83.9) | 20 (16.1) | 2.26 (1.28-4.01) | **0.005** | 1.63 (0.81-3.29) | 0.171 |
| No | 506 (92.2) | 43 (7.8) | 1.00 |  | 1.00 |  |
| **Reported history of high cholesterol** |  |  |  |  |  |  |
| No checkup | 814 (91.6) | 75 (8.4) | 2.18 (0.67-7.09) | 0.195 | 2.93 (0.80-10.71) | 0.104 |
| Yes | 14 (93.3) | 1 (6.7) | 1.69 (0.16-17.46) | 0.659 | 1.87 (0.16-21.76) | 0.618 |
| No | 71 (95.9) | 3 (8.1) | 1.00 |  | 1.00 |  |

Abbreviations: AOR, adjusted odds ratio; CI, confidence interval; COR, crude odds ratio; DBP, diastolic blood pressure; SBP; systolic blood pressure; SD, standard deviation

^a^ WHO global recommendations on physical activity
